# Highly syntenic regions in the genomes of soybean, *Medicago truncatula*, and *Arabidopsis thaliana*

**DOI:** 10.1186/1471-2229-5-15

**Published:** 2005-08-15

**Authors:** Joann Mudge, Steven B Cannon, Peter Kalo, Giles ED Oldroyd, Bruce A Roe, Christopher D Town, Nevin D Young

**Affiliations:** 1 Dept of Plant Pathology, 495 Borlaug Hall, University of Minnesota, St. Paul, MN 55108 USA; 2 Dept. of Disease and Stress Biology, John Innes Centre, Norwich Research Park, Colney Norwich, NR4 7UH, UK; 3 The Advanced Center for Genome Technology (ACGT), Stephenson Research & Technology Center, University of Oklahoma, Norman OK 73019 USA; 4 The Institute for Genomic Research (TIGR), 9712 Medicago Center Drive, Rockville, MN 20850 USA

## Abstract

**Background:**

Recent genome sequencing enables mega-base scale comparisons between related genomes. Comparisons between animals, plants, fungi, and bacteria demonstrate extensive synteny tempered by rearrangements. Within the legume plant family, glimpses of synteny have also been observed. Characterizing syntenic relationships in legumes is important in transferring knowledge from model legumes to crops that are important sources of protein, fixed nitrogen, and health-promoting compounds.

**Results:**

We have uncovered two large soybean regions exhibiting synteny with *M. truncatula *and with a network of segmentally duplicated regions in *Arabidopsis*. In all, syntenic regions comprise over 500 predicted genes spanning 3 Mb. Up to 75% of soybean genes are colinear with *M. truncatula*, including one region in which 33 of 35 soybean predicted genes with database support are colinear to *M. truncatula*. In some regions, 60% of soybean genes share colinearity with a network of *A. thaliana *duplications. One region is especially interesting because this 500 kbp segment of soybean is syntenic to two paralogous regions in *M. truncatula *on different chromosomes. Phylogenetic analysis of individual genes within these regions demonstrates that one is orthologous to the soybean region, with which it also shows substantially denser synteny and significantly lower levels of synonymous nucleotide substitutions. The other *M. truncatula *region is inferred to be paralogous, presumably resulting from a duplication event preceding speciation.

**Conclusion:**

The presence of well-defined *M. truncatula *segments showing orthologous and paralogous relationships with soybean allows us to explore the evolution of contiguous genomic regions in the context of ancient genome duplication and speciation events.

## Background

The rapid increase in eukaryotic genome sequence in recent years enables genome-wide alignments, megabase (Mb)-scale comparisons between species, and fine-scaled phylogenetic footprinting. Recent sequenced-based studies in a variety of organisms have described high levels of synteny (conservation of gene content and order between species) within kingdoms and between families, but have also highlighted frequent synteny loss and degradation due to gene duplication, deletion, and rearrangement. In some cases, observed synteny has been extensive. In vertebrates, over 90% of the mouse and human genomes (separated by 91 million years; My) lie in syntenic blocks [1,2], some exceeding 40 Mb [2,3]. At a greater evolutionary distance (310 My), the human and chicken genomes show large synteny blocks, including at least 70 Mb of highly conserved sequence [2,4]. Regions syntenic to 1.8 Mb of human DNA were identified in twelve different species including fish, which separated from humans 450 Mya [2,5].

High levels of synteny have also been found in plant families. Molecular marker analysis has allowed chromosome-by-chromosome alignments of several genera within the Solanaceae, Fabaceae, and Poaceae [6-8]. Generally, syntenic relationships are complicated by micro- and macro-rearrangements as well as duplications [9]. Complete genome sequences of rice and *A. thaliana*, models representing the two major clades of flowering plants, allows comparisons across a greater evolutionary distance. Separated by 200 My, rice and *Arabidopsis thaliana *nonetheless retain substantial conserved syntenic blocks, including one region spanning 119 *A. thaliana *genes [10].

Though genomic relationships within legumes are less well characterized, a growing number of studies have begun to reveal extensive synteny between the members of this important plant family. Based on restriction fragment length polymorphisms (RFLPs), substantial genome conservation was discovered among Phasoloid species, including mungbean (*Vigna radiata*) and cowpea (*V. unguiculata*), extending as long as entire chromosomes [11]. Comparable levels of synteny were later demonstrated between Vigna and the common bean, *Phaseolus vulgaris *[12]. Synteny with the more distant soybean, *Glycine max*, was more limited, typically on the order of 10 – 20 cM. Later, Lee et al. [13] observed higher levels of conservation between bean, mungbean, and soybean, where *A. thaliana *also showed conservation to some conserved legume regions and even helped to elucidate duplicated regions in soybean. Choi et al. [6] described genome-wide macrosynteny among legumes using a large set of cross-species genetic markers. Though genomic correspondence was reduced by chromosomal rearrangements increasing with phylogenetic distance, they could align chromosomes from a variety of Papilionoid species, including *Medicago truncatula *and soybean.

*M. truncatula *and *Lotus japonicus *are two model legumes that are now targets of large-scale genome sequencing. With more than 100 Mb of genome sequence publicly available in both, genome-scale comparisons at both the macro- and micro-syntenic level are possible. Young et al [14] compared all finished and anchored sequence between these two genomes (111 Mb) and concluded that more than 75% of both genomes reside in conserved, syntenic segments. At a microsyntenic scale, Choi et al. [6] analyzed ten BAC/TAC clone pairs and found 80% of genes were conserved and colinear. Soybean has also been compared to *M. truncatula *because of its economic importance. With few sequences 100 kbp or more in length available, however, comparisons of soybean with reference legumes have been limited to low resolution surveys and short contiguous segments. Nevertheless, conserved synteny is widespread between *M. truncatula *and soybean. Yan et al. [15] analyzed three homologous BAC contig groups in detail by comparative physical mapping and cross-hybridization and found six of eight genome regions exhibited conserved synteny, including three that were extensively conserved. In genome-wide survey of synteny, slightly more than half of 50 RFLP-based soybean BAC-contigs, each approximately 200 kbp in size, exhibited conserved synteny with *M. truncatula *[16] and nearly 75% of these cases were extensive.

**Figure 1:**
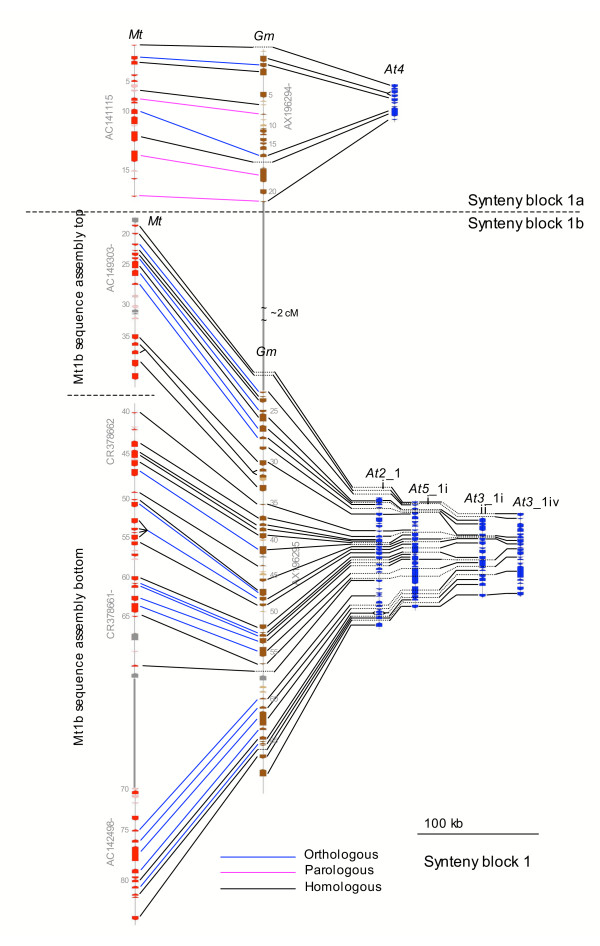
Synteny block 1. A syntenic block of soybean, *M. truncatula *and *A. thaliana *genes surrounding soybean's *rhg1 *gene (Gm gene 21). Solid black lines connect homologs. Dotted black lines indicate that the absence of a homolog in the syntenic position. Blue lines connect orthologs. Pink lines connect paralogs. *M. truncatula *genes are shown in red, soybean in brown, and *A. thaliana *in blue. Lighter colored genes represent those that had no significant similarity to Genbank's nonredundant protein database. Gray genes are repetitive elements. A thick gray vertical line connecting sequence assemblies indicate regions in which sequence is not yet available but in which linkage and approximate distance were determined. Genbank accessions are shown in gray.

**Figure 2:**
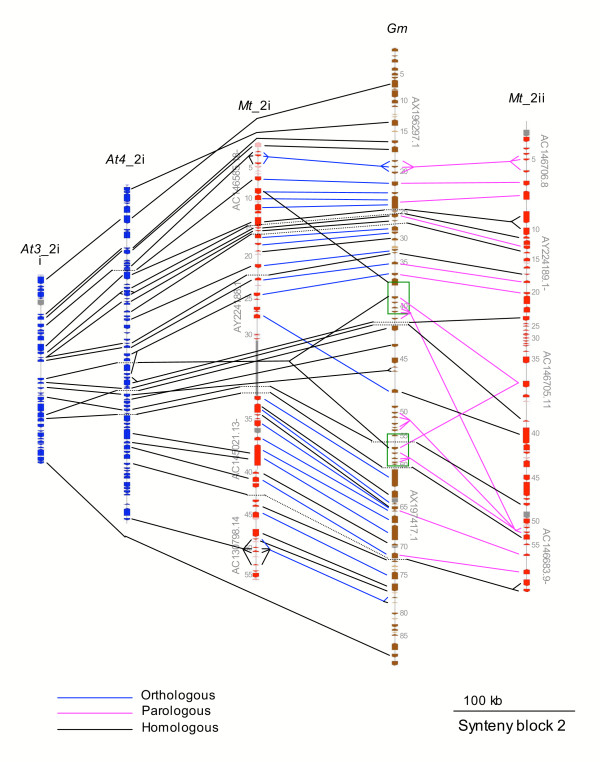
Synteny block 2. A syntenic block of soybean, *M. truncatula *and *A. thaliana *sequence assemblies surrounding soybean's *Rhg4 *gene (Gm gene 29). Solid black lines connect homologs. Blue lines connect orthologs. Pink lines connect paralogs. Dotted black lines indicate that the absence of a homolog in the syntenic position. *M. truncatula *genes are shown in red, soybean in brown, and *A. thaliana *in blue. Lighter colored genes represent those that had no significant similarity to Genbank's nonredundant protein database. Gray genes are repetitive elements. A thick gray line connecting sequence assemblies indicate regions in which sequence is not yet available but in which physical linkage and approximate physical distance were determined. Numbers along the sequence assemblies indicate gene numbers. Genbank accessions are shown in gray. Green boxes identify a 25 kbp duplication.

In the course of our genome sequencing work in *M. truncatula *[14], two regions were observed to be significantly conserved with previously sequenced regions of soybean. These soybean regions contain two important soybean cyst nematode (SCN) (*Heterodera glycines*) resistance loci, *rhg1 *and *Rhg4*, which have been studied extensively reviewed in Concibido, et al. [17]. In previous work, our lab and others localized the genetic positions of these genes and characterized their role in resistance [18-24]. We saturated the regions with genetic markers, developed high throughput molecular markers, created physical maps, and characterized homoeologous and surrounding genome regions [23,25-30]. As a result of the extensive information available and the importance of SCN resistance, these genome regions were eventually sequenced [31,32], including the tentative cloning of *rhg1 *and *Rhg4 *(gene 29 in Figure 1 and gene 21 in Figure 2, respectively).

A preliminary examination of the soybean *rhg1 *region described in the present study concluded that nearly 70% of genes were conserved and colinear between soybean and *M. truncatula *[6]. Previously, Foster-Hartnett et al. [29] had used survey sequences along a 1 Mb stretch that included and extended beyond the region described here to examine syntenic relationships with *A. thaliana*. Based on survey (primarily BAC-end) sequence that included both genic and non-genic regions, 35% of soybean sequences were conserved in one or more syntenic *A. thaliana *regions. The soybean region formed a network of synteny with six different *A. thaliana *regions. The longest syntenic segment in *A. thaliana *was more than 2 Mb in length, highlighting the existence of long stretches of conserved sequence between distantly related genomes [29].

In the present study, we describe the gene content in two soybean regions totaling approximately 1 Mb in size with more than 150 genes [32] that exhibit extensive synteny to *M. truncatula *and *A. thaliana*. The two soybean regions reside on different chromosomes but are functionally linked – each contains a receptor-like kinase gene (*rhg1 *or *Rhg4*) tentatively identified as a resistance gene to SCN (*Heterodera glycines*). Up to 75% of soybean genes in this region are colinear with *M. truncatula*, including one 300 kbp segment with 33 of 35 soybean genes colinear to *M. truncatula*. Nearly 60% of the genes in this same soybean region exhibit colinearity with one or more *A. thaliana *regions. These highly syntenic blocks are discussed in the context of phylogenetic data revealing patterns of evolution and orthologous and paralogous relationships.

## Results

### Identifying and mapping homologous contigs

We used the Genbank soybean sequences surrounding SCN resistance loci as a basis for searching all available *M. truncatula *BAC sequences and the *A. thaliana *proteome for syntenic regions (Table 1). Homologous regions were then used to create corresponding sequence assemblies for all three genera (Figures 1, 2). Where possible, we also merged sequence *M. truncatula *assemblies by identifying end-sequenced BACs that spanned gaps between non-overlapping BACs. Most of the *M. truncatula *BACs could be anchored to the *M. truncatula *genetic map (Table 1) [33].

There is a gap in all three species between synteny blocks 1a and 1b which we were unable to span. In soybean, these two sequence assemblies are genetically linked and located 2 cM apart on LG-G. In addition, synteny block 1b contained two gaps in *M. truncatula *(Figure 1), one a 90 kbp gap toward the bottom *M. truncatula *sequence assembly. This gap and its surrounding *M. truncatula *sequence (totaling about 175 kbp) correspond to an insertion/deletion in soybean just 25 kbp in size and containing two gene models without hits to Genbank's nonredundant (nr) database along with one repetitive sequence. There is also a gap of unknown size between the top and bottom sequence assemblies in synteny block 1b that could not be spanned with end sequenced BACs. In *M. truncatula*, the top sequence assembly maps to *M. truncatula *chromosome 4, while the bottom maps to chromosome 3 (Table 1). These two *M. truncatula *assemblies therefore appear to be unlinked, even though they show substantial synteny and are apparently both orthologous (see below) to a contiguous region in soybean.

*M. truncatula *sequences in synteny block 2 also map to chromosomes 3 and 4. One of the *M. truncatula *homoeologs in synteny block 2, *Mt*_2ii, maps 5–8 cM below the *M. truncatula *bottom sequence assembly in synteny block 1b (Figure 1, 2, Table 1). The other *M. truncatula *duplicate in synteny block 2, *Mt_*2i, maps to chromosome 4, more than 25 cM from the top sequence assembly in synteny block 1b (Figure 1, 2, Table 1).

### Synteny

#### Soybean/*Medicago truncatula *synteny within synteny block 1

The soybean and *M. truncatula *regions in synteny block 1b are highly syntenic, with nearly complete conservation of orientation and order of conserved genes (Table 2, Figure 1). Seventy-five percent of soybean genes in this region (33 of 44) have *M. truncatula *homologs and 59% of *M. truncatula *genes (33 of 56) have soybean homologs. When this comparison is revised to include only genes with significant matches to Genbank's nr database (thereby eliminating potentially poor gene calls) even more extensive levels of synteny emerge: 33 of 35 soybean genes with nr hits (94%) have *M. truncatula *homologs conserved in order and orientation. The two soybean genes without *M. truncatula *homologs include the *rhg1 *resistance locus itself, as well as one hypothetical protein. Both of these genes along with a soybean gene that extends beyond available *M. truncatula *sequence data all show synteny to *A. thaliana*. Therefore, all 36 confirmed soybean genes in this 336 kbp region have homologs in syntenic regions either of *M. truncatula *or *A. thaliana*.

Synteny block 1a shows lower, yet still impressive synteny. Nearly half of the genes in synteny block 1a are conserved in order and orientation. Indeed, 44% of *M. truncatula *genes are conserved in order and orientation in soybean in block 1a, increasing to 50% when only genes with database support are considered. Of soybean genes, 37% are conserved in *M. truncatula*, increasing to 43% of genes with database support.

#### Soybean/*Medicago truncatula *synteny in synteny block 2

Like block 1, extensive synteny is also evident throughout synteny block 2 (Figure 2, Table 2). In synteny block 2, there are two duplicated regions of *M. truncatula *syntenic to soybean, *Mt*_2i and *Mt*_2ii, which flank the soybean segment in Figure 2. The *Mt*_2i homoeolog and soybean share 60% (28 of 47) of their genes. With two exceptions, orientation is conserved between *Mt*_2i and soybean, and remarkably, a run of 13 out of 13 confirmed soybean genes are perfectly conserved in *Mt*_2i in the bottom portion of block 2. The corresponding soybean region extends nearly 110 kbp (Figure 2).

The other *M. truncatula *homoeolog, *Mt*_2ii, shows synteny with soybean extending more than 300 kbp (Figure 2, Table 2). In this region, soybean shares 32% (12 of 38) of genes and M. truncatula homoeolog *Mt*_2ii, 24% (12 of 50) in this syntenic region. One gene, with similarity to a rapid alkalinization factor in *Solanum chacoense*, shows synteny between soybean and *Mt*_2ii but appears to have been lost from *Mt*_2i (Figure 2, *Gm *gene 34, *Mt*_2ii gene 18). The middle portion of synteny block 2 exhibits multiple rearrangements and duplications between soybean and *Mt*_2ii (Figure 2). While much of the corresponding *Mt*_2i region has not yet been sequenced, it is less than half the size of the rearranged region in *Mt*_2ii/soybean on the basis of BAC-end sequenced clones that span the *Mt*_2i region.

The *Mt*_2i and *Mt*_2ii homoeologs themselves share nine genes, only one of which is absent from soybean (Figure 2). The gene absent from soybean encodes a putative AMP-binding protein (Figure 2, *Mt*_2i gene 14, *Mt*_2ii genes 9–10) present in one copy in *Mt*_2i and two adjacent copies in *Mt*_2ii. By contrast, *Mt*_2i and soybean share three times as many homologous pairs as the two *M. truncatula *duplicates themselves, including 19 homologous pairs that are absent from *Mt*_2ii. These observations help to illuminate the orthologous and paralogous relationships of these genome regions, which are described in further detail below.

#### Comparisons with *A. thaliana*

High levels of synteny are also maintained between the two legume species and networks of duplicated *A. thaliana *regions, each with a unique pattern of gene loss (Table 2). For example, nearly 62% (28 of 45) soybean genes and half of *M. truncatula *genes in synteny block 1b have a homolog within a syntenic network of four *A. thaliana *duplicated regions (Figure 1). With any one *A. thaliana *region, much lower levels of conserved synteny are observed (between 23% and 34% of soybean genes; 17% and 27% of *M. truncatula *genes). These results are consistent with the model of large-scale genome duplication followed by gene loss in *Arabidopsis *[34] and mirror the results of Foster-Hartnett et al. [29] in their low resolution synteny analysis of the soybean *rhg1 *region. By contrast, we found only one region in *A. thaliana *syntenic to block 1a (Figure 1, Table 2) with 20% (4 of 20) of soybean genes and 29% (5 of 17) of *M. truncatula *genes. In synteny block 2, levels of synteny between the legume species and individual *A. thaliana *regions were comparable to those in synteny blocks 1a and 1b, but composite syntenies were much lower (Table 2). For instance, just 29% of soybean genes were conserved in *At4*_2i and 17% in *At3*_2ii, with only 31% conserved in the network of both *A. thaliana *regions.

### Perspectives on cyst nematode resistance genes

Despite the absence of an *rhg1 *homolog in the syntenic *M. truncatula *region examined here (synteny block 1b, *Mt*, top sequence assembly in Figure 1), it is clear that there is an *rhg1 *homolog elsewhere in *M. truncatula*. Though not present in any of the full-length BAC sequences that currently comprise >40% of *M. truncatula's *genespace [33], a homolog does exist on a *M. truncatula *BAC-end sequence (mth2-60m7), showing 78% amino acid identity to *rhg1 *over 279 amino acids (e-value = e-118). This percent identity is higher than that of the syntenic *M. truncatula *homologs compared to soybean's *Rhg4 *locus in synteny block 2 (~70%). BAC mth2-60m7 and the next three BAC-end hits all belong to the same region of *M. truncatula *FPC contig 949 [33], which tentatively maps to chromosome 5. This indicates that either the *M. truncatula rhg1 *homolog was translocated out of the remainder of the syntenic region on chromosome 4 or that contig 949 represents a paralogous region with the *rhg1 *homolog lost from the orthologous region that we examined here. Given the extensive synteny that exists in the region surrounding *rhg1*, it is surprising that the *M. truncatula *homolog of this gene, in particular, has undergone such rearrangement and/or loss. Notably, a homolog to rhg1 does exist among the network of *A. thaliana *duplicates (Figure 1, Synteny block 1b, *At3*_1iii, gene 5).

On the other hand, homologs of *Rhg4 *were found in the corresponding regions of *M. truncatula *and *A. thaliana*, including both *M. truncatula *homoeologs (Figure 2, *Mt_*2i gene 8, *Mt_*2ii gene 7, and *At3*_2ii gene 11). The *M. truncatula Rhg4 *homologs both show approximately 70% identity with *Rhg4 *and the surrounding region is greatly conserved as well. *Mt*_2i shares 75% and *Mt*_2ii 47%, of confirmed genes with soybean in the region immediately surrounding the *Rhg4 *homologs. *Mt*_2i even shows conservation of 100% of confirmed genes in a region over 300 kbp away. The high conservation of genome context surrounding *Rhg4 *indicates that, had the *M. truncatula *sequence been available before *Rhg4 *was cloned, it would have greatly facilitated cross genomic chromosome walking and cloning of the *Rhg4 *gene.

### Tandem duplications

Genes have undergone tandem duplication in all species (soybean: 7 genes, *M. truncatula*: 9 genes, *A. thaliana*: 6 genes). In four cases, homologous soybean/*M. truncatula *genes are both duplicated. In no cases are homologous *A. thaliana*/legume genes both duplicated.

Tandemly duplicated genes with the highest copy numbers occur in a highly rearranged region in the middle of synteny block 2 (Figure 2). The rearranged region in soybean contains 11 copies of chalcone synthase genes in three separate groups of four, four, and three genes (genes 39–42, 50–51, 53–54, 57–59 in Figure 2). The latter group appears to have originated from a 25 kbp segmental duplication of the top CHS group and surrounding genes. While soybean has 11 copies of the CHS gene in this region, including CHS1, CHS2, CHS3, CHS4, and CHS5, the *Mt*_2ii region has only one CHS cluster with two genes, CHS1A and CHS1B (genes 52–53 in Figure 2). In addition, *Mt*_2ii contains a group of 10 genes with similarity to *A. thaliana *auxin-induced proteins 6B and X10A that are absent in soybean (genes 24–33 in Figure 2). It was not possible to analyze the corresponding region in *Mt*_2i, as this genome segment has not yet been sequenced.

Tandem duplications occur in other regions as well (Figures 1, 2). There are examples of tandemly duplicated genes whose homolog(s) are not duplicated, as well as cases in which two or more homologs have duplicated. For example, soybean and both *M. truncatula *duplicates in synteny block 2 have three copies of a glucosyltransferase (*Mt*_2i genes 2–4, *Gm *genes 18–20, and *Mt_*2ii genes 4–6 in Figure 2). Cases of differential tandem duplication may have resulted from duplication in only one species or loss of duplicates from one species.

### Phylogenetic analysis

Phylogenetic trees were successfully generated for 21 gene families with members in synteny block 1 and for 23 in synteny block 2, many of which included homologs from both *M. truncatula *duplicates (Figures 1, 2) [see Additional Files 1, 2]. These phylogenies were examined to determine whether soybean and *M. truncatula *homologs within synteny blocks were more closely related to each other than to homologs elsewhere in the genomes, as represented by expressed sequences. For all 16 phylogenies in synteny block 1b, *M. truncatula*/soybean homologs were more closely related to each other than to homologs in other genomic regions, strongly suggesting orthology (Figure 1) [see Additional File 1]. Synteny block 1a contained a mix of tentative orthologs (two comparisons) and paralogs (three comparisons) (Figure 1) [see Additional File 1]. In synteny block 2, soybean genes showed orthologous relationships with their homologs in *M. truncatula *block *Mt*_2i every time (20 of 20 comparisons) and paralogous relationships in *M. truncatula *homoeolog *Mt*_2ii (11 of 11 comparisons) (Figure 2) [see Additional File 2].

**Figure 3:**
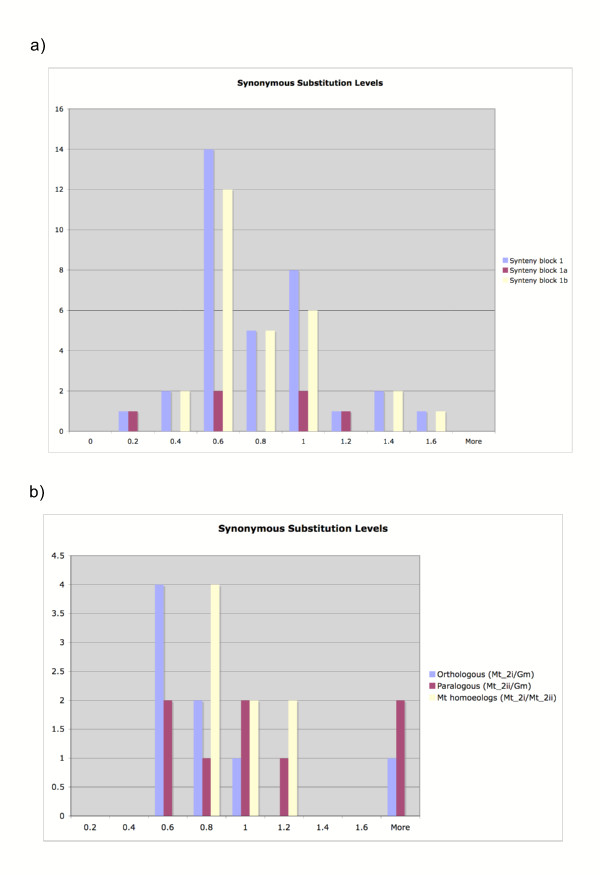
Histograms of synonymous distance between soybean and *M. truncatula *homologs or *M. truncatula *homoeologs in a) synteny block 1 and b) synteny block 2. For synteny block 2, only the 8 genes with homologs in soybean and both *M. truncatula *duplicates are shown.

Nucleotide substitution levels were determined to measure the evolutionary distance between soybean and *M. truncatula *(synonymous substitution levels) and to identify differences, if any, in selection pressure (nonsynonymous substitution levels). In synteny block 1, estimates of synonymous and nonsynonymous substitution levels were obtained for 34 sets of *M. truncatula *and soybean homologs (Figure 3a), six in synteny block 1a and 28 in synteny block 1b. In comparing these two blocks, we observed no difference in the number of synonymous substitutions per site (Table 3; 1a: 0.71, 1b: 0.71, p = 0.96), suggesting similar times of divergence between soybean and *M. truncatula *in both regions. This result is somewhat surprising given the fact that block 1a is composed of a mixture of apparent orthologous and paralogous relationships, while block 1b exhibits exclusively orthologous relationships.

In synteny block 2, the two *M. truncatula *homoeologs shared the same eight genes with soybean. We therefore focused on paired comparisons using these eight genes. The extent of synonymous substitutions between soybean and *Mt*_2i (0.87), an orthologous relationship based on phylogenetic tree analysis, was significantly lower than the extent between soybean and *Mt*_2ii (1.21), a paralogous relationship (p = 0.008) (Table 3; Figure 3b). All eight paired comparisons show higher levels of synonymous substitutions in the paralogous comparison. Not surprisingly, therefore, the paralogous region has evolved farther from soybean in evolutionary time than the orthologous region, implying that a duplication spanning the entire synteny block 2 preceded speciation between *M. truncatula *and soybean. The number of nonsynonymous substitution levels per site were comparable between the orthologous and paralogous *M. truncatula*/soybean relationships, with no significant difference (p = 0.69) (Table 3).

Comparisons of the distance between the two *M. truncatula *homoeologs in synteny block 2 revealed levels of synonymous substitutions (0.82) comparable to those of the orthologous *Mt*_2i/soybean comparison in the same block (0.87) (Table 3; Figure 3b; p = 0.85), suggesting that the duplication may have occurred close in time with speciation. It is surprising that the synonymous distance between *M. truncatula *homoeologs (0.82) is not closer to that of the paralogous *M. truncatula*/soybean comparison (1.21), which should be comparable given a duplication event followed by speciation. However, the difference between them was not significant (p = 0.22). There were no significant differences when comparing homoeologs between synteny blocks (data not shown) for either synonymous or nonsynonymous substitution levels. No differences were observed between tandemly duplicated and single copy genes for nonsynonymous or synonymous substitution levels (data not shown).

Estimates of synonymous substitution distance between orthologous soybean and *M. truncatula *regions allowed us to estimate the time of the *Medicago*/*Glycine *speciation event, as did the synonymous distance between the two *M. truncatula *duplicates in synteny block 2 in timing the underlying genome duplication. Orthologous regions between soybean and *M. truncatula *in both synteny blocks 1b (*Mt/Gm*) and 2 (*Mt*_2i/*Gm*) (Figures 1, 2) give similar estimates of divergence since speciation. Synteny block 1b has a median synonymous substitution level of 0.61 per site (Table 3), suggesting 50 My since the divergence through speciation, using an estimate of 6.1 × 10^-9 ^substitutions per synonymous site per year [35]. Synteny block 2 has a median synonymous substitution level of 0.59 per site (Table 3) when comparing all orthologs, suggesting 48 Mya since speciation. By contrast, the duplication event in *M. truncatula *evident in synteny block 2 (Figure 2) appears to have predated speciation, with a median of 0.79 synonymous substitutions per site and an inferred divergence between duplicates of 64 Mya.

## Discussion

### Synteny

#### Soybean and *M. truncatula *synteny

We examined two regions that are highly syntenic between soybean, *M. truncatula*, and *A. thaliana*. These two regions comprise approximately 0.5 Mb each surrounding the *rhg1 *and *Rhg4 *SCN resistance loci of soybean and their corresponding regions in *M. truncatula *and *A. thaliana*. In the process, we discovered remarkably high levels of colinearity between soybean and *M. truncatula*, including cases of near perfect conservation of gene order and orientation. For example, we observed one case where 33 of 35 genes and a second where 13 out of 13 genes were perfectly conserved and colinear. Because *M. truncatula *and soybean are estimated to have diverged approximately 50 Mya, these examples of conserved synteny are truly remarkable. Overall, soybean synteny to *M. truncatula *in orthologous relationships averages 79% and reaches 94% when only genes with Genbank's nonredundant database support are considered. Moreover, synteny between the legume species and a network of *A. thaliana *segmental duplications exceeds 60%.

Levels of synteny are clearly not this high genome-wide, though macrosynteny exists over much of the genome [6]. Yan et al. [16] estimated that synteny between soybean and *M. truncatula *exists in only about half of soybean genomic regions anchored by RFLPs, and of these cases, just 75% exhibit extensive synteny. In other genome regions not described in here, we have found much lower levels of synteny. In a targeted search for syntenic relationships between soybean and *M. truncatula *in genome regions surrounding the soybean disease resistance gene, *rpg1*, Cannon et al. [36] discovered much lower levels of synteny. The best syntenic candidate regions that could be identified showed significant differential gene expansion, multiple rearrangements, indels, and translocations. In this case, the *rpg1 *soybean region on linkage group F of approximately 300 kbp corresponded to an *M. truncatula *region of more than 500 kbp. Of 30 soybean genes and 45 *M. truncatula *genes confirmed by hits to the Uniref database [37] at BLASTP, e ≤ -4, just nine (20%) were syntenic.

Why the regions that we examined in the current study should have remained so highly conserved is unknown. Several explanations for differential conservation of synteny have been proposed. Regions with disease resistance genes often evolve rapidly and show frequent rearrangements [38-40]. Nevertheless, the soybean regions in this study were characterized because they contain important disease resistance genes – though not members of the more widespread NBS-LRR gene family most frequently associated with rearrangements [38]. Often, regions near centromeres tend to be more conserved than telomeric regions [41]. But the soybean region in synteny block 1 is known to be located at the very end of the chromosome, while still retaining high levels of synteny with *M. truncatula *and *A. thaliana*. Of the mapped *M. truncatula *sequence assemblies, only the top sequence assembly in synteny block 1b is close to the centromere (R. Geurts et al., personal communication). In some organisms, regions of housekeeping genes are clustered and thought to be more conserved [42-44]. Though housekeeping genes are certainly present in the regions of this study, the regions do not represent clusters of housekeeping genes [see Additional File 3]. Finally, the presence of transposons and other repetitive sequence may decrease stability in a region [45-47]. In the regions described here, just 12 of 356 predicted gene models in soybean and *M. truncatula *are transposons or retrotransposons. On a base pair level, this translates to 2.2% of *M. truncatula *and 1.7% of soybean genomic sequence. These levels of transposons and retrotransposons are not unusual. Cannon et al. (personal communication) estimated that transposons of this type occupy ~3% of the *M. truncatula *genome when examining over 100 Mb of sequence. In soybean, Graham et al. [48] found that more than 10% of a soybean BAC containing multiple NBS-LRR sequences was composed of retrotransposons. Although we cannot identify hallmarks of the sequences examined that would cause them to be more conserved than usual, they do appear to be highly conserved and care should be taken in drawing general conclusions from this comparison.

#### Legume and *A. thaliana *synteny

The relationship between legumes and *A. thaliana *in synteny block 1b, described in detail here, seem to follow a pattern of post-speciation duplication followed by gene loss [34]. Several syntenic regions exist that vary slightly in overall degree of synteny, while gene loss/insertion has occurred in every case. A composite of all the partially syntenic regions come together to form a network that together recapitulates substantial genome conservation. The retention of soybean and *M. truncatula *genes in *A. thaliana *is impressive, given the roughly 90 My thought to separate *A. thaliana *from legumes [49]. For example, synteny block 1b has 60% of its genes occurring in at least one of four syntenic *A. thaliana *regions, though individually, the most conserved of these regions contains just half that level.

Though synteny between legumes and *A. thaliana *in this region is impressive, previous results suggest it extends beyond the region analyzed here. Foster-Hartnett et al [29] described conserved synteny involving the genome region around *rhg1 *twice the size examined in the present study, though at low sequence resolution primarily using BAC-end sequences [29]. Simillion, et al. [50] also found conservation among the *A. thaliana *regions syntenic to synteny block 1b extending up to 182 kbp in length.

#### Exceptions to synteny

There were also some consistent exceptions to synteny. A total of 13 transposons, including retrotransposons, were found among the three species. Just one was found in syntenic positions, where an *M. truncatula *retroelement in synteny block 1b was located in a comparable position to an element in soybean. However, the soybean (a copia-type polyprotein) and *M. truncatula *(an RNA-directed DNA polymerase) genes do not share sequence homology.

Additionally, genes predicted by FGENESH but without database support were much less likely to have homologs than those with database confirmation (9% of soybean predicted genes without database support versus 62% of those with database support have homologs). Still, a small number – roughly 10% – of nearly 60 unconfirmed genes (no hits in nr) in either soybean or *M. truncatula *did show synteny. Conserved legume genes without homologs in the database may be the most interesting genes of all, since they are likely to be novel or highly diverged from known proteins and may play a role in important plant or legume-specific processes.

### Gene density

Although soybean's genome size is more than double that of *M. truncatula *[51], gene density is comparable in these regions (Synteny block 1: *M. truncatula *= 1 gene/7.2 kb, soybean: 1 gene/6.7 kb; Synteny block 2: *M. truncatula *= 1 gene/6.2 kb, soybean: 1 gene/5.8 kb). These values are not far from those of *A. thaliana *overall (1 gene/5 kb) [52]. In this region, *A. thaliana *gene density is approximately half to two-thirds that of the legumes (Synteny block 1: *A. thaliana *= 1 gene/3.4 kb; Synteny block 2: *A. thaliana *= 1 gene/4.1 kb), although soybean and *M. truncatula *genome sizes are 7X and 3X that of *A. thaliana*, respectively [51]. Higher than expected gene densities in soybean and *M. truncatula *suggest the possibility of gene clustering. Indeed, gene clustering has identified in *M. truncatula *[53] and forms the basis of targeting *M. truncatula *sequencing to the gene-rich regions of the genome [14].

### Genome duplication and speciation

The networks of synteny we have identified reflect duplication and gene loss between species, including *M. truncatula *regions with both orthologous and paralogous relationships to soybean. In synteny block 2, for example, there are clear-cut examples of regions with orthologous and paralogous relationships. *Mt*_2i shows only orthologous relationships with soybean, while *Mt*_2ii shows only paralogous relationships (Figures 1, 2). *M. truncatula *regions in synteny block 1b also unambiguously display orthology to soybean.

*M. truncatula *duplications in synteny block 2 allowed us to systematically examine corresponding orthologous and paralogous relationships to soybean. The percentage of conserved genes between soybean and *Mt*_2i (orthologous region) was twice as high as with *Mt*_2ii (paralogous region) (Figure 2, Table 2). Given that orthology indicates the most closely related regions evolutionarily (reflected in the phylogenetic trees) [see Additional File 2], it is not surprising that fewer genes have been deleted/inserted or experienced substitutions in orthologous comparisons. The fact that all the *M. truncatula *genes in the orthologous region (*Mt*_2i) are more closely related to soybean as evidenced by phylogenetic trees, synonymous substitution levels, percent identity, and extent of synteny, than either is to the *M. truncatula *genes in the paralogous region (*Mt*_2ii) suggests that the duplication seen in *M. truncatula *occurred before the speciation event splitting *Medicago *and *Glycine *lineages. Presumably, soybean also has (or had) a duplicate region as well, a possibility with some phylogenetic support [see Additional File 2].

We date the duplication event, possibly as a part of a genome duplication, in *M. truncatula *at 64 Mya, preceding a speciation event approximately 48–50 Mya. Indeed, the possibility of a genome duplication event predating the split between *M. truncatula *and soybean has been suggested previously [16,54,55]. Median synonymous substitution levels between the two *M. truncatula *duplicates in synteny block 2 (0.79 synonymous substitutions per site) fall within [55] or near [54] synonymous distance peaks, which were interpreted by the authors as a genome duplication event in *M. truncatula*. Schleuter et al. [55] estimates that this event occurred 58 million years ago, while Blanc and Wolfe [54] inferred a more recent event based on a substantially different molecular clock [56]. Likewise, we estimate the speciation event between *Medicago *and *Glycine *at 48 – 50 Mya, while Blanc and Wolfe [54] inferred a much more recent date of 13.3–15 million years ago, though again, the differences are primarily due to the use of differing molecular clocks [56].

Comparatively long (~500 kbp) and contiguous sets of homologous segments from different species with known phylogenetic relationships and nucleotide substitution levels bring power to the study of molecular evolution. Though median synonymous substitution levels of duplication and speciation events correspond well to published values [54,55] (see above), the extent of synonymous substitutions varies significantly between neighboring genes despite a common genomic context (Figure 3). Estimates comparing the two *M. truncatula *segments created by a duplication event range from 0.62–1.12 while those comparing soybean and *M. truncatula *orthologs (speciation event) range from 0.42–2.68. Since the duplicates in each one of these cases presumably diverged at the same moment, one must postulate different evolutionary trajectories for the different gene lineages. Knowing that all the genes on a contiguous genomic block duplicated (and later speciated) together removes an important unknown from evolution analyses in contrast to comparable EST-based studies [54,55].

## Conclusion

We analyzed genome regions of soybean, *M. truncatula*, and *A. thaliana *with remarkable levels of conservation of gene content and order. Such high levels of colinearity within the legumes and with the model plant *A. thaliana *bode well for leveraging information from model genomes to crop plants like soybean. Further, we described substantial blocks of genes with the same evolutionary (duplication) history, allowing us to study and compare the individual evolution of genes within a common genomic context. These blocks include two duplicates in *M. truncatula*, one orthologous and the other paralogous with soybean. This duplication may be part of a larger genome duplication event in the common ancestor of soybean and *M. truncatula*. If so, the analysis described here is just the first step in understanding the evolution of legume genomes and a useful addition to our knowledge about genomic reorganization that occurs at a the scale of megabase or less.

## Methods

### Sequences

*Glycine max *sequences [GenBank:AX196294.1, GenBank:AX196295.1, GenBank:AX196297.1, and GenBank:AX197417.1] [32] were obtained from Genbank. All soybean sequences were derived from cultivar 'A3244'. [GenBank:AX196295.1] (Figure 1, gene 29) includes the susceptible allele of soybean *rhg1 *gene on molecular linkage group G. [GenBank:AX197297] (Figure 2, gene 21) includes the susceptible allele of soybean *Rhg4 *gene on molecular linkage group A2.

*M. truncatula *BACs were sequenced as part of an international effort to sequence the genespace of this model legume [14]. Two additional *M. truncatula *BACs were sequenced and examined before the international genome sequencing had begun [57]. Putative homologs of soybean sequences in *M. truncatula *and *A. thaliana *were identified by searching the soybean sequences against all sequenced *M. truncatula *BACs and the *A. thaliana *proteome using BLAST [58] (The Institute for Genomic Research, *Arabidopsis *Proteome version 5). After identifying genes (see below), protein/protein comparisons (BLASTP) were performed in order to confirm that BACs were syntenic and to identify syntenic genes (see below). Genbank accessions for soybean and *M. truncatula *sequences, *A. thaliana *gene numbers, and mapping information are shown in Table 1.

### Sequence assemblies

Sequences were aligned and merged in regions of sequence overlap on the basis of 99% identity or better. End-sequenced BAC clones that tentatively spanned gaps in the sequence were identified based on strong hits (e-value = 0, ≥99% identity) to sequenced BACs on either side. Gap sizes were estimated by removing overlap from the estimated size of end-sequenced BAC(s).

### Nomenclature

Throughout the manuscript, the following nomenclature is used. Regions surrounding and syntenic to the SCN resistance *rhg1 *locus are collectively referred to as synteny block 1 (Figure 1). Regions surrounding and syntenic to SCN *Rhg4 *gene are collectively referred to as synteny block 2 (Figure 2). Synteny block 1 is divided into blocks 1a and 1b, which are separated by gaps in all three species (Figure 1). Within each synteny block, species are labeled as Gm (soybean), *Mt *(*M. truncatula*), or At (*A. thaliana*). The chromosome number follows the "At" abbreviation for *A. thaliana*. If more than one homoeolog is present, the species abbreviation is appended with an underscore followed by the synteny block and lower case roman numerals (i.e. *Mt*_2i, *Mt*_2ii, *At4*_2i, and *At3*_2ii in Figure 2) (Figures 1, 2). Sequence assemblies separated by physical gaps are labeled as sequence assemblies "top" and "bottom" in arbitrary order (Figure 1).

### Gene prediction and identification of synteny

Genes were predicted in *G. max *and *M. truncatula *genomic sequences using the dicot (*Arabidopsis*) matrix of FGENESH [59,60]. BLASTP was used to compare predicted proteins between databases containing these *G. max *or *M. truncatula *predicted genes and all *A. thaliana *proteins with an e-value cutoff of e-8 and percent identity cutoff of 40% for the top high scoring segment pair for soybean and *M. truncatula *comparisons and an e-value cutoff of e-8 for comparisons to *A. thaliana *[58]. These cutoff values generally identified homologs in syntenic positions while rejecting related genes in nonsyntenic positions.

In this study, we defined synteny to include both conservation of gene content and order between species. In estimating syntenic density (the percentage of genes conserved between two species), repetitive sequences (genes with similarity to transposable elements, including retroelements) were not included and tandemly duplicated genes were counted as one. Synteny between two species was estimated from the first to the last pair of conserved genes in the available sequence for both species.

### Phylogenetic analysis

To distinguish between orthologous and paralogous regions, we constructed phylogenetic trees as follows. BLASTP or TBLASTN, as appropriate, were used to compare all *G. max *genes with the following sequences: all *G. max *and *M. truncatula *proteins in the corresponding genomic regions of this analysis; the nonredundant *A. thaliana *proteome; soybean and *M. truncatula *EST unigene sets [61] (GMGI v.11 and *MT*GI v.7; The Institute for Genomic Research. Rockville, MD). The top 25 hits ≥100 amino acids with e-values ≤ e-10 were included in the analysis. Tandem duplications and highly related genes in the same gene family were grouped for analysis.

Initial alignments were calculated using T-COFFEE [62] with manual evaluations and edits in Jalview [63] for poorly aligning sequences. For subsequent phylogenetic analysis, an HMM calculated for each alignment using hmmer [64] was used to realign sequences and to identify and remove indel regions and sequences with fewer than 60% matches to the model. Parameters for hmmbuild were: archpri = 0.7, gapmax = 0.3.

Parsimony trees were calculated using the protpars of Phylip [65], with maximum likelihood branch lengths calculated using TREE-PUZZLE [66]. Parameters for protpars were: randomize input order; use ordinary parsimony; search for best tree; select one best tree for further analysis in TreePuzzle. Parameters for TreePuzzle were: user defined tree (from parsimony search); approximate parameter estimates; Whelan-Goldman substitution model [67] estimate amino acid frequencies from data set; allow rate heterogeneity with eight gamma-distributed rates.

### Nucleotide substitutions

Codon-aligned nucleic acid sequences were created with TranslateAlign.pl (courtesy Dan Kortschak, University of Adelaide, Adelaide, Australia). Nucleotide substitutions levels were calculated using these alignments with SNAP (Synonymous/Non-synonymous Analysis Program) [68,69]. In this program, the levels of synonymous and nonsynonymous substitutions per site are approximated using methods developed by Nei and Gojobori [70], incorporating Ota and Nei's statistic [71]. Median synonymous substitution levels were converted into estimates of time since divergence using an estimate of 6.1 × 10^-9 ^substitutions per synonymous site per year [35].

## Abbreviations

Megabases (Mb); kilobase pairs (kbp); million years (My); million years ago (Mya); soybean cyst nematode (SCN); restriction fragment length polymorphism (RFLP); *Medicago truncatula *(Mt); *Glycine max *(Gm); *Arabidopsis thaliana *(At)

## Authors' contributions

JM carried out the synteny, phylogenetic, and nucleotide substitution analyses and drafted the manuscript. SBC participated in the design of the study, the phylogenetic analysis, and the interpretation of the data. PK and GEDO participated in the sequencing, including identification of BACs in the area of interest. BAR participated in the sequencing. CDT participated in the interpretation of data and writing of the manuscript. NDY participated in the design, analysis, and interpretation of the data as well as in the writing of the manuscript. All authors read and approved the final manuscript.

## Supplementary Material

Additional File 1 Phylogenetic trees of genes in synteny block 1. For soybean and *M. truncatula*, genes are labeled with their homoeolog name and gene number as in Figure 1. *A. thaliana *genes are labeled by their standard gene numbers. Other genes represent expressed sequences. Scales are in PAM units. Click here for file

Additional File 2 Phylogenetic trees of genes in synteny block 2. For soybean and *M. truncatula*, genes are labeled with their homoeolog name and gene number as in Figure 2. *A. thaliana *genes are labeled by their standard gene numbers. Other genes represent expressed sequences. Scales are in PAM units. Click here for file

Additional File 3 Gene Annotation Table Click here for file

## Figures and Tables

**Table 1:** Sequence accession, contig, and map positions

Synteny Block	Species	Homoeolog	Sequence assembly	Genbank accession or *Arabidopsis *gene numbers^1^	Orientation	Contig^2^	length (kbp)	chromosome	Position^3^	Anchoring marker
1a	*M. truncatula*	Mt		AC141115.22	+	246	126			
	
1a	Soybean	Gm		AX196294.1	-		127	G	5.8	B053_1^4^
	
1a	*A. thaliana*	At4			+		30	4	18.4	

1b	*M. truncatula*	Mt	top	AC149303.10	-	945	141	4	32.5	DK379L^5,7^
1b	*M. truncatula*	Mt	bottom	CR378662.1^a^	+	1072	119	3	59	
1b	*M. truncatula*	Mt	bottom	CR937029.1^a^	-	1072	112	3	59	h2_105b15c, h2_6m1c^6^
1b	*M. truncatula*	Mt	bottom	AC142498.20	-	1072	114	3	59	
	
1b	Soybean	Gm		AX196295.1	+		336	G	3.9–4.5	Sat_168, Satt309^4^
	
1b	*A. thaliana*	At2_1i		At2g40130-At2g40400	-		107	2	16.8	
1b	*A. thaliana*	At5_1ii		At5g05350-At5g05600	+		89	5	1.6	
1b	*A. thaliana*	At3_1iii		At3g55970-At3g56140	-		66	3	20.8	
1b	*A. thaliana*	At3_1iv		At3g10980-At3g11180	+		69	3	3.4	

2	*M. truncatula*	*Mt*_2i		AC146585.18^b^	-	273	125	4	58.7	
2	*M. truncatula*	*Mt*_2i		AY224188.1^b^	+		99	4	58.7	
2	*M. truncatula*	*Mt*_2i		AC145021.13^c^	-	114	120	4	58.7	EST948^5^
2	*M. truncatula*	*Mt*_2i		AC130798.14^c^	+	114	92	4	58.7	
	
2	Soybean			AX196297.1^d^	+		350	A2	48.8	I^4^
2	Soybean			AX197417.1^d^	+		214	A2	48.8	I^4^
	
2	*M. truncatula*	*Mt*_2ii		AC146706.8^e^	+	1132	107	3	64.2	h2_108g5a^6^
2	*M. truncatula*	*Mt*_2ii		AY224189.1^e^	-		68	3	67.6	AY_224189_a^6^
2	*M. truncatula*	*Mt*_2ii		AC146705.11^e^	+	1132	120	3	63.5	h2_101f3d^6^
2	*M. truncatula*	*Mt*_2ii		AC146683.9^e^	-	1132	131	3	63.5	
	
2	*A. thaliana*	At4_2i		At4g13600-At4g14200	-		278	4	7.9	
2	*A. thaliana*	At3_2ii		At3g23670-At3g24000	+		156	3	8.5	

^1 ^Letters denote groups of sequence accessions that overlap

^2 ^Physical contig number [33]

^3 ^Measured in cM for soybean and M. truncatula and in Mb for A. thaliana

^4 ^[72]

^5 ^[33]

^6 ^Denny et al., unpublished

^7 ^Marker is from BAC Mth2-31m16, which is adjacent in the contig but not overlapping

**Table 2:** Synteny

Synteny Block	Reference species	Homoeolog	Syntenic Species	Homoeolog	Synteny (% genes)	Synteny (# of genes)	Total Genes^1^	Synteny (% confirmed genes)	Synteny (# of confirmed genes)	Total Confirmed Genes^1^
1a	Soybean		*M. truncatula*		37%	7	19	43%	6	14
1a	*M. truncatula*		Soybean		44%	7	16	50%	6	12
1a	Soybean		*A. thaliana*		20%	4	20			
1a	*M. truncatula*		*A. thaliana*		29%	5	17			
1a	*A. thaliana*		*Soybean*		50%	4	8			
1a	*A. thaliana*		*M. truncatula*		50%	5	10			

1b	Soybean		*M. truncatula*		75%	33	44	94%	33	35
1b	*M. truncatula*		Soybean		59%	33	56	79%	33	42
1b	Soybean		*A. thaliana*	Composite At2_1i-At3_1iv	62%	28	45			
				At2_1i	34%	14	41			
				At5_1ii	25%	11	44			
				At3_1iii	25%	10	40			
				At3_1iv	23%	7	30			
1b	*M. truncatula*		*A. thaliana*	Composite At2_1i-At3_1iv	50%	28	56			
				At2_1i	27%	13	48			
				At5_1ii	21%	12	56			
				At3_1iii	17%	9	52			
				At3_1iv	24%	8	33			

2	Soybean		*M. truncatula*	*Mt*_2i	60%	28	47	66%	27	41
2	*M. truncatula*	*Mt*_2i	Soybean		60%	28	47	72%	26	36
2	Soybean		*M. truncatula*	*Mt*_2ii	32%	12	38	33%	12	36
2	*M. truncatula*	*Mt*_2ii	Soybean		24%	12	50	26%	12	46
2	Soybean		*M. truncatula*	Composite *Mt*_2i, *Mt*_2ii	66%	31	47	73%	30	41
2	Soybean		*A. thaliana*	Composite	31%	20	64			
2	Soybean		*A. thaliana*	At4_2i	29%	16	56			
2	Soybean		*A. thaliana*	At3_2ii^2^	17%	11	64			
2	*M. truncatula*	*Mt*_2i	*A. thaliana*	Composite At4_2i, At3_2ii	26%	12	47			
2	*M. truncatula*	*Mt*_2i	*A. thaliana*	At4_2i	23%	11	47			
2	*M. truncatula*	*Mt*_2i	*A. thaliana*	At3_2ii	27%	6	22			
2	*M. truncatula*	*Mt*_2ii	*A. thaliana*	Composite At4_2i, At3_2ii	37%	15	41			
2	*M. truncatula*	*Mt*_2ii	*A. thaliana*	At4_2i	32%	13	41			
2	*M. truncatula*	*Mt*_2ii	*A. thaliana*	At3_2ii	22%	8	36			

^1 ^Calculated from first to last syntenic gene in region

^2 ^If the last syntenic pair is removed, 10 of 35 genes are syntenic (29%)

**Table 3:** Extent of Nucleotide Substitution between Soybean and *M. truncatula *(Mean/Median)

Synteny Block	Homoeologs	Nonsynonymous/Synonymous^1,2^	Synonymous substitutions^1,3^	Nonsynonymous substitutions^1,4^
1	Mt and Gm	^a ^0.42/0.27	^a ^0.71/0.73	^a ^0.19/0.23
1	Mt and Gm	^a ^0.21/0.18	^a ^0.71/0.61	^a ^0.13/0.12
2^5^	*Mt*_2i and Gm	^a,b ^0.26/0.25	^a ^0.87/0.63	^a ^0.16/0.12
2^5^	*Mt*_2ii and Gm	^a ^0.17/0.16	^b ^1.21/0.98	^a ^0.17/0.15
2^5^	*Mt*_2i and *Mt*_2ii	^b ^0.24/0.24	^a,b ^0.82/0.79	^b ^0.21/0.21

^1 ^Letters denote significant difference at p ≤ 0.05

^2 ^Ratio of synonymous to nonsynonymous substitutions

^3 ^Synonymous substitutions per site

^4 ^Nonsynonymous substitutions per site

^5 ^Synteny block 2 includes only the eight genes in soybean and both *M. truncatula *duplicates

## References

[B1] Mouse Genome Sequencing Consortium (2002). Initial sequencing and comparative analysis of the mouse genome. Nature.

[B2] Hedges SB (2002). The origin and evolution of model organisms. Nat Rev Genet.

[B3] Zhao S, Shetty J, Hou L, Delcher A, Zhu B, Osoegawa K, de Jong P, Nierman WC, Strausberg RL, Fraser CM (2004). Human, mouse, and rat genome large-scale rearrangements: Stability versus speciation. Genome Res.

[B4] International Chicken Genome Sequencing Consortium (2004). Sequence and comparative analysis of the chicken genome provide unique perspectives on vertebrate evolution. Nature.

[B5] Thomas JW, Touchman JW, Blakesley RW, Bouffard GG, Beckstrom-Sternberg SM, Margulies EH, Blanchette M, Siepel AC, Thomas PJ, McDowell JC, Maskeri B, Hansen NF, Schwartz MS, Weber RJ, Kent WJ, Karolchik D, Bruen TC, Bevan R, Cutler DJ, Schwartz S, Elnitski L, Idol JR, Prasad AB, Lee-Lin SQ, Maduro VV, Summers TJ, Portnoy ME, Dietrich NL, Akhter N, Ayele K, Benjamin B, Cariaga K, Brinkley CP, Brooks SY, Granite S, Guan X, Gupta J, Haghighi P, Ho SL, Huang MC, Karlins E, Laric PL, Legaspi R, Lim MJ, Maduro QL, Masiello CA, Mastrian SD, McCloskey JC, Pearson R, Stantripop S, Tiongson EE, Tran JT, Tsurgeon C, Vogt JL, Walker MA, Wetherby KD, Wiggins LS, Young AC, Zhang LH, Osoegawa K, Zhu B, Zhao B, Shu CL, De Jong PJ, Lawrence CE, Smit AF, Chakravarti A, Haussler D, Green P, Miller W, Green ED (2003). Comparative analyses of multi-species sequences from targeted genomic regions. Nature.

[B6] Choi HK, Mun JH, Kim DJ, Zhu H, Baek JM, Mudge J, Roe B, Ellis N, Doyle J, Kiss GB, Young ND, Cook DR (2004). Estimating genome conservation between crop and model legume species. Proc Natl Acad Sci U S A.

[B7] Moore G, Devos KM, Wang Z, Gale MD (1995). Cereal genome evolution. grasses, line up and form a circle. Curr Biol.

[B8] Tanksley SD, Ganal MW, Prince JP, de Vicente MC, Bonierbale MW, Broun P, Fulton TM, Giovannoni JJ, Grandillo S, Martin GB (1992). High density molecular linkage maps of the tomato and potato genomes. Genetics.

[B9] Salse J, Piegu B, Cooke R, Delseny M (2004). New in silico insight into the synteny between rice (oryza sativa L.) and maize (zea mays L.) highlights reshuffling and identifies new duplications in the rice genome. Plant J.

[B10] Goff SA, Ricke D, Lan TH, Presting G, Wang R, Dunn M, Glazebrook J, Sessions A, Oeller P, Varma H, Hadley D, Hutchison D, Martin C, Katagiri F, Lange BM, Moughamer T, Xia Y, Budworth P, Zhong J, Miguel T, Paszkowski U, Zhang S, Colbert M, Sun WL, Chen L, Cooper B, Park S, Wood TC, Mao L, Quail P, Wing R, Dean R, Yu Y, Zharkikh A, Shen R, Sahasrabudhe S, Thomas A, Cannings R, Gutin A, Pruss D, Reid J, Tavtigian S, Mitchell J, Eldredge G, Scholl T, Miller RM, Bhatnagar S, Adey N, Rubano T, Tusneem N, Robinson R, Feldhaus J, Macalma T, Oliphant A, Briggs S (2002). A draft sequence of the rice genome (Oryza sativa L. ssp. japonica). Science.

[B11] Menanciohautea D, Fatokun CA, Kumar L, Danesh D, Young ND (1993). Comparative genome analysis of mungbean (*Vigna-radiata *l-wilczek) and cowpea (*V-unguiculata*l walpers) using RFLP mapping data. Theoretical & Applied Genetics.

[B12] Boutin SR, Young ND, Olson TC, Yu ZH, Shoemaker RC, Vallejos CE (1995). Genome conservation among three legume genera detected with DNA markers. Genome.

[B13] Lee JM, Grant D, Vallejos CE, Shoemaker RC (2001). Genome organization in dicots. II. *Arabidopsis *as a 'bridging species' to resolve genome evolution events among legumes. Theoretical & Applied Genetics.

[B14] Young NevinD, Cannon StevenB, Shusei Sato, Dongjin Kim, Cook DouglasR, Town ChrisD, Roe BruceA, Satoshi Tabata (2005). Sequencing the genespaces of *Medicago truncatula *and *Lotus japonicus*. Plant Physiology.

[B15] Yan HH, Mudge J, Kim DJ, Shoemaker RC, Cook DR, Young ND (2004). Comparative physical mapping reveals features of microsynteny between *Glycine max, Medicago truncatula*, and *Arabidopsis thaliana*. Genome.

[B16] Yan HH, Mudge J, Kim DJ, Larsen D, Shoemaker RC, Cook DR, Young ND (2003). Estimates of conserved microsynteny among the genomes of *Glycine max*, *Medicago truncatula *and *Arabidopsis thaliana*. Theor Appl Genet.

[B17] Concibido VC, Diers BW, Arelli PR (2004). A decade of QTL mapping for cyst nematode resistance in soybean. Crop Science.

[B18] Concibido VC, Denny RL, Boutin SR, Hautea R, Orf JH, Young ND (1994). DNA marker analysis of loci underlying resistance to soybean cyst nematode (*Heterodera glycines *ichinohe). Crop Science.

[B19] Concibido VC, Denny RL, Lange DA, Orf JH, Young ND (1996). RFLP mapping and marker-assisted selection of soybean cyst nematode resistance in PI 209332. Crop Science.

[B20] Concibido VC, Young ND, Lange DA, Denny RL, Danesh D, Orf JH (1996). Targeted comparative genome analysis and qualitative mapping of a major partial-resistance gene to the soybean cyst nematode. Theoretical & Applied Genetics.

[B21] Concibido VC, Lange DA, Denny RL, Orf JH, Young ND (1997). Genome mapping of soybean cyst nematode resistance genes in Peking, PI 90763, and PI 88788 using DNA markers. Crop Science.

[B22] Meksem K, Doubler TW, Chancharoenchai K, Njiti VN, Chang SJC, Arelli APR, Cregan PE, Gray LE, Gibson PT, Lightfoot DA (1999). Clustering among loci underlying soybean resistance to *Fusarium solani*, SDS and SCN in near-isogenic lines. Theoretical & Applied Genetics.

[B23] Meksem K, Pantazopoulos P, Njiti VN, Hyten LD, Arelli PR, Lightfoot DA (2001). 'Forrest' resistance to the soybean cyst nematode is bigenic: Saturation mapping of the *Rhg1 *and *Rhg4 *loci. Theoretical & Applied Genetics.

[B24] Webb DM, Baltazar BM, Raoarelli AP, Schupp J, Clayton K, Keim P, Beavis WD (1995). Genetic mapping of soybean cyst nematode race-3 resistance loci in the soybean PI 437.654. Theoretical & Applied Genetics.

[B25] Cregan PB, Mudge J, Fickus EW, Danesh D, Denny R, Young ND (1999). Two simple sequence repeat markers to select for soybean cyst nematode resistance conditioned by the *rhg1 *locus. Theoretical & Applied Genetics.

[B26] Meksem K, Zobrist K, Ruben E, Hyten D, Quanzhou T, Zhang HB, Lightfoot DA (2000). Two large-insert soybean genomic libraries constructed in a binary vector: Applications in chromosome walking and genome wide physical mapping. Theoretical & Applied Genetics.

[B27] Meksem K, Ruben E, Hyten DL, Schmidt ME, Lightfoot DA (2001). High-throughput genotyping for a polymorphism linked to soybean cyst nematode resistance gene *Rhg4 *by using TaqMan (TM) probes. Molecular Breeding.

[B28] Meksem K, Ruben E, Hyten D, Triwitayakorn K, Lightfoot DA (2001). Conversion of AFLP bands into high-throughput DNA markers. Mol Genet Genomics.

[B29] Foster-Hartnett D, Mudge J, Larsen D, Danesh D, Yan H, Denny R, Penuela S, Young ND (2002). Comparative genomic analysis of sequences sampled from a small region on soybean (*Glycine max*) molecular linkage group G. Genome.

[B30] Danesh D, Penuela S, Mudge J, Denny RL, Nordstrom H, Martinez JP, Young ND (1998). A bacterial artificial chromosome library for soybean and identification of clones near a major cyst nematode resistance gene. Theoretical & Applied Genetics.

[B31] Clough SJ, Tuteja JH, Li M, Marek LF, Shoemaker RC, Vodkin LO (2004). Features of a 103-kb gene-rich region in soybean include an inverted perfect repeat cluster of CHS genes comprising the *I *locus. Genome.

[B32] Hauge BM, Wang ML, Parsons JD, Parnell LD Nucleic acid molecules and other molecules associated with soybean cyst nematode resistance. Patent: WO 0151627-A 2 19-JUL-2001.

[B33] *Medicago truncatula *sequencing resources. http://www.medicago.org/genome/.

[B34] Ku HM, Vision T, Liu J, Tanksley SD (2000). Comparing sequenced segments of the tomato and arabidopsis genomes: Large-scale duplication followed by selective gene loss creates a network of synteny. Proc Natl Acad Sci U S A.

[B35] Lynch M, Conery JS (2000). The evolutionary fate and consequences of duplicate genes. Science.

[B36] Cannon S, Makarevich G, Savage E, Denny R, Mudge J, Seigfried M, Lai H, Ashfield T, Roe BA, Young ND, Innes R (2005). A phylogenetic and structural comparison of homologous *Rpg1 *R-gene-containing regions in soybean and *Medicago truncatula*. Plant and Animal Genomes XIII: San Diego, CA.

[B37] Apweiler R, Bairoch A, Wu CH, Barker WC, Boeckmann B, Ferro S, Gasteiger E, Huang H, Lopez R, Magrane M, Martin MJ, Natale DA, O'Donovan C, Redaschi N, Yeh LS (2004). UniProt: The universal protein knowledgebase. Nucleic Acids Res.

[B38] Baumgarten A, Cannon S, Spangler R, May G (2003). Genome-level evolution of resistance genes in *Arabidopsis thaliana*. Genetics.

[B39] Leister D, Kurth J, Laurie DA, Yano M, Sasaki T, Devos K, Graner A, Schulze-Lefert P (1998). Rapid reorganization of resistance gene homologues in cereal genomes. Proc Natl Acad Sci U S A.

[B40] Michelmore RW, Meyers BC (1998). Clusters of resistance genes in plants evolve by divergent selection and a birth-and-death process. Genome Res.

[B41] Akhunov ED, Akhunova AR, Linkiewicz AM, Dubcovsky J, Hummel D, Lazo G, Chao S, Anderson OD, David J, Qi L, Echalier B, Gill BS, Miftahudin, Gustafson JP, La Rota M, Sorrells ME, Zhang D, Nguyen HT, Kalavacharla V, Hossain K, Kianian SF, Peng J, Lapitan NL, Wennerlind EJ, Nduati V, Anderson JA, Sidhu D, Gill KS, McGuire PE, Qualset CO, Dvorak J (2003). Synteny perturbations between wheat homoeologous chromosomes caused by locus duplications and deletions correlate with recombination rates. Proc Natl Acad Sci U S A.

[B42] Chuang JH, Li H (2004). Functional bias and spatial organization of genes in mutational hot and cold regions in the human genome. PLoS Biol.

[B43] Zhang L, Li W (2004). Mammalian housekeeping genes evolve more slowly than tissue-specific genes. Mol Biol Evol.

[B44] Lercher MJ, Urrutia AO, Hurst LD (2002). Clustering of housekeeping genes provides a unified model of gene order in the human genome. Nat Genet.

[B45] Hua-Van A, Daviere JM, Kaper F, Langin T, Daboussi MJ (2000). Genome organization in *Fusarium oxysporum*: Clusters of class II transposons. Curr Genet.

[B46] Zhang J, Peterson T (1999). Genome rearrangements by nonlinear transposons in maize. Genetics.

[B47] Zhang J, Peterson T (2004). Transposition of reversed Ac element ends generates chromosome rearrangements in maize. Genetics.

[B48] Graham MA, Marek LF, Shoemaker RC (2002). Organization, expression and evolution of a disease resistance gene cluster in soybean. Genetics.

[B49] Gandolfo MA, Nixon KC, Crepet WL (1998). A new fossil flower from the turonian of new jersey – *Dressiantha bicarpellata *gen. et sp. nov. (capparales). American Journal of Botany.

[B50] Simillion C, Vandepoele K, Saeys Y, Van de Peer Y (2004). Building genomic profiles for uncovering segmental homology in the twilight zone. Genome Res.

[B51] Royal Botanic Gardens, Kew: Plant DNA C-values Database (release 3.0, December 2004). http://www.rbgkew.org.uk/cval/homepage.html.

[B52] The Arabidopsis Genome Initiative (2000). Analysis of the genome sequence of the flowering plant arabidopsis thaliana. Nature.

[B53] Kulikova O, Gualtieri G, Geurts R, Kim DJ, Cook D, Huguet T, de Jong JH, Fransz PF, Bisseling T (2001). Integration of the FISH pachytene and genetic maps of medicago truncatula. Plant J.

[B54] Blanc G, Wolfe KH (2004). Widespread paleopolyploidy in model plant species inferred from age distributions of duplicate genes. Plant Cell.

[B55] Schlueter JA, Dixon P, Granger C, Grant D, Clark L, Doyle JJ, Shoemaker RC (2004). Mining EST databases to resolve evolutionary events in major crop species. Genome.

[B56] Koch MA, Haubold B, Mitchell-Olds T (2000). Comparative evolutionary analysis of chalcone synthase and alcohol dehydrogenase loci in *Arabidopsis*, *Arabis*, and related genera (Brassicaceae). Mol Biol Evol.

[B57] Zhu H, Kim DJ, Baek JM, Choi HK, Ellis LC, Kuester H, McCombie WR, Peng HM, Cook DR (2003). Syntenic relationships between *Medicago truncatula *and *Arabidopsis *reveal extensive divergence of genome organization. Plant Physiol.

[B58] Altschul SF, Gish W, Miller W, Myers EW, Lipman DJ (1990). Basic local alignment search tool. J Mol Biol.

[B59] Salamov A, Solovyev V (2000). *Ab initio *gene finding in drosophila genomic DNA. Genome Research.

[B60] SoftBerry – fgenesh. http://sun1.softberry.com/berry.phtml?topic=fgenesh&group=programs&subgroup=gfind.

[B61] Quackenbush J, Cho J, Lee D, Liang F, Holt I, Karamycheva S, Parvizi B, Pertea G, Sultana R, White J (2001). The TIGR gene indices: Analysis of gene transcript sequences in highly sampled eukaryotic species. Nucleic Acids Res.

[B62] Notredame C, Higgins DG, Heringa J (2000). T-coffee: A novel method for fast and accurate multiple sequence alignment. J Mol Biol.

[B63] Clamp M, Cuff J, Searle SM, Barton GJ (2004). The Jalview java alignment editor. Bioinformatics.

[B64] Eddy SR (1998). Profile hidden markov models. Bioinformatics.

[B65] Felsenstein J (2000). PHYLIP (Phylogeny Inference Package) Version 3.6. Distributed by the Author.

[B66] Schmidt HA, Strimmer K, Vingron M, von Haeseler A (2002). TREE-PUZZLE: Maximum likelihood phylogenetic analysis using quartets and parallel computing. Bioinformatics.

[B67] Goldman N, Whelan S (2000). Statistical tests of gamma-distributed rate heterogeneity in models of sequence evolution in phylogenetics. Mol Biol Evol.

[B68] Korber B, Rodrigo AG, Learn GH (2000). Signature and sequence variation analysis. Computational Analysis of HIV Molecular Sequences.

[B69] HIV Sequence Database: SNAP Submission Form. http://www.hiv.lanl.gov/content/hiv-db/SNAP/WEBSNAP/SNAP.html.

[B70] Nei M, Gojobori T (1986). Simple methods for estimating the numbers of synonymous and nonsynonymous nucleotide substitutions. Mol Biol Evol.

[B71] Ota T, Nei M (1994). Variance and covariances of the numbers of synonymous and nonsynonymous substitutions per site. Mol Biol Evol.

[B72] Song QJ, Marek LF, Shoemaker RC, Lark KG, Concibido VC, Delannay X, Specht JE, Cregan PB (2004). A new integrated genetic linkage map of the soybean. Theor Appl Genet.

